# Blue mussels of the *Mytilus edulis* species complex from South America: The application of species delimitation models to DNA sequence variation

**DOI:** 10.1371/journal.pone.0256961

**Published:** 2021-09-02

**Authors:** Pablo A. Oyarzún, Jorge E. Toro, José J. Nuñez, Elkin Y. Suárez-Villota, Jonathan P. A. Gardner

**Affiliations:** 1 Centro de Investigación Marina Quintay (CIMARQ), Universidad Andres Bello, Quintay, Chile; 2 Facultad de Ciencias Universidad Austral de Chile, Instituto de Ciencias Marinas y Limnológicas (ICML), Valdivia, Chile; 3 Facultad de Medicina Veterinaria y Agronomía, Instituto de Ciencias Naturales (ICCNN), Universidad de las Américas, Concepción, Chile; 4 School of Biological Sciences, Victoria University of Wellington, Wellington, New Zealand; Laboratoire de Biologie du Développement de Villefranche-sur-Mer, FRANCE

## Abstract

Smooth-shelled blue mussels, *Mytilus* spp., have a worldwide antitropical distribution and are ecologically and economically important. Mussels of the *Mytilus edulis* species complex have been the focus of numerous taxonomic and biogeographical studies, in particular in the Northern hemisphere, but the taxonomic classification of mussels from South America remains unclear. The present study analysed 348 mussels from 20 sites in Argentina, Chile, Uruguay and the Falkland Islands on the Atlantic and Pacific coasts of South America. We sequenced two mitochondrial locus, Cytochrome *c* Oxidase subunit I (625 bp) and 16S rDNA (443 bp), and one nuclear gene, ribosomal 18S rDNA (1770 bp). Mitochondrial and nuclear loci were analysed separately and in combination using maximum likelihood and Bayesian inference methods to identify the combination of the most informative dataset and model. Species delimitation using five different models (GMYC single, bGMYC, PTP, bPTP and BPP) revealed that the *Mytilus edulis* complex in South America is represented by three species: native *M*. *chilensis*, *M*. *edulis*, and introduced Northern Hemisphere *M*. *galloprovincialis*. However, all models failed to delimit the putative species *Mytilus platensis*. In contrast, however, broad spatial scale genetic structure in South America using Geneland software to analyse COI sequence variation revealed a group of native mussels (putatively *M*. *platensis*) in central Argentina and the Falkland Islands. We discuss the scope of species delimitation methods and the use of nuclear and mitochondrial genetic data to the recognition of species within the *Mytilus edulis* complex at regional and global scales.

## Introduction

Species delimitation, i.e. the act of identifying boundaries at the species level [1,2], is necessary for systematics, ecology and evolution, and fundamental to the accurate assessment of biodiversity as well as for implementing conservation policies. Often this act can be relatively easy owing to allopatry or prezygotic barriers to reproduction, but in many cases species delimitation is made difficult by the presence of cryptic variation and the limitations of many species concepts to effectively recognise such entities [3,4]. In recent years, there has been an increase in the number of methods for delimiting species [5–10], some of which attempt to provide a robust theoretical and repeatable analytical framework for species identification. Species delimitation methods usually examine molecular variation data (usually DNA sequences) and utilise phylogenetic reconstructions to statistically identify a threshold that distinguishes species from populations. The accuracy of the method depends largely on the rate of speciation, the population size and the genetic variation used (number of informative sites or loci). These delimitation approaches remove the subjectivity associated with an individual researcher’s view of what constitutes a species, and have been helpful in solving taxonomic problems, particularly those of closely related species (e.g., Lemer et al. [11]), but are not yet widely applied.

Marine bivalve mussels of the genus *Mytilus* are naturally distributed throughout all continents of the world excluding Antarctica [12], and as common members of the intertidal community they play important roles in energy transfer from the pelagic to the benthic realm [13,14]. The smooth-shelled blue mussel *Mytilus edulis* species complex consists of three widely recognised and closely related species: *Mytilus edulis* Linné, 1758, *M*. *galloprovincialis* Lamarck, 1819 and *M*. *trossulus* Gould, 1850 [13]. However, there has been longstanding debate about the existence of other smooth-shelled blue mussel species, dating back over 100 years (refer to [15–17] for reviews and to Hilbish et al. [14]; Gérard et al. [18] for more recent molecular interpretations). This is true for all of the Southern hemisphere, but particularly so for the Atlantic and Pacific coasts of South America, despite a large body of work addressing the subject in regions such as Chile (e.g., [18–28] and references therein). To the best of our knowledge, models of species delimitation have not been applied to the *Mytilus edulis* species complex problem, although they may have the ability to resolve the global taxonomy of this widespread and important group.

In South America, blue mussels occur naturally from Dichato, Chile (36°32′S; 72°56′ W) on the Pacific coast, around Cape Horn (55°58′ S; 67°17′ W), and extend north along the Atlantic coastline to a northern limit at Punta del Este, Uruguay (34°58′S; 54°57′W) [29]. The distribution of these animals also includes the Patagonian and Atlantic Ocean islands (e.g. Tierra del Fuego and the Falkland Islands). These blue mussels are important members of the benthic fauna of the South American coast [30], and are an important resource for aquaculture in the region, in particular, in Chile [31]. The Atlantic coast blue mussel was originally described by d’Orbigny [32], based on morphometric grounds, as *M*. *platensis*. Only eight years subsequently, again based on morphometric grounds, the Pacific coast blue mussel was described by Hupé [33] as *M*. *chilensis*. Since the advent of genetic markers, a range of marker types (with or without associated morphometric analyses) have been applied to native South American mussels to address the question of their taxonomy. Variously, South American mussels have been described as *M*. *edulis*-like [13], as *M*. *edulis platensis* [24], as *M*. *platensis* [26,34], as a Southern hemisphere lineage of *Mytilus galloprovincialis* also found in other Pacific Ocean regions such as New Zealand and Australia [22,23,35], as *Mytilus edulis chilensis* [36,37], and as *Mytilus chilensis* [28,38–43]. Several authors have noted that different marker types and analyses of different genomes (i.e., mitochondrial versus nuclear DNA) may provide different answers, and that newer generations of marker types, increased genomic coverage, and also wider geographic sampling may be required to definitively answer the question of which species occur where (e.g., McDonald et al. [13], Oyarzún et al. [41], Larraín et al. [43]). In addition, specifically relating to the situation on the Pacific coast of South America, it was noted (e.g., Borsa et al. [24], Oyarzún et al. [27]) that at the time the name *M*. *chilensis* had no formal standing, despite its widespread usage. This situation has changed recently, and WoRMS now lists *M*. *chilensis* Hupé, 1854 as having “accepted” species status. For native mussels on the Atlantic Ocean coast of South America, WoRMS continues to recognise the valid status of *M*. *platensis* d’Orbigny, 1846. This fluidity of taxonomic status highlights the challenge of working with a species complex, the advances in taxonomy that new molecular markers can provide, and the difficulty of achieving a taxonomic outcome that is both biologically relevant and accepted by workers in the field.

The development of species-specific nuclear and mitochondrial DNA RFLP assays to blue mussels from many parts of the world has substantially improved our understanding of the phylogeography and specific status of members of the smooth-shelled blue mussel complex [19,23,39,44–51]. However, the situation in the Southern hemisphere is still much less clear than that in the Northern hemisphere, and in need of further attention for three reasons. First, delimitation of species allows clarification of the taxonomy in a stable and consistent way. This is of importance because the species is the fundamental unit in biology and is used for activities such biodiversity classification, biosecurity monitoring and breeding programmes [52–55]. Second, clarification of the taxonomy and systematics permits a better understanding of the conservation threats posed by invasive mussels and the consequences of hybridisation and introgression on the genetic integrity of native mussels [27,34,43]. Third, species delimitation may have important implications in other areas, such as food production (e.g. aquaculture), where strict regulations exist to protect consumer rights and for reasons of traceability around which species may be grown, moved within a country or between countries, sold on the local market, or exported (i.e. European Normative, Regulation (CE) N° 104/2000 and N° 2065/2001 –[39,43,56–58]. In this context, blue mussels form the basis of mussel aquaculture industries in many countries [59,60] so that accurate species determination is important, both in terms of traceability and marketing [43,61,62].

The aim of the present research was to address the question of the taxonomic status of blue mussels from the Atlantic and Pacific coasts of South America, and thereby to better understand the phylogeography of the taxa identified. For context, such work needs to be carried out with reference to other *Mytilus* species elsewhere. We use both nuclear and mitochondrial DNA sequence variation to delimit putative native and introduced taxa in the full distributional range of *Mytilus* spp. in South America–Chile, Argentina, Uruguay and the Falkland Islands. The uncertain taxonomy of the *Mytilus* spp. system is used to test speciation hypotheses with different species delimitation methods, in an attempt to move beyond qualitative assessments of population-specific or lineage/taxon-specific genetic differences. We view this first application of species delimitation models to the South American blue mussels as a case study which, if successful, may then be applied to the global situation using existing and/or new genetic data sets.

## Materials and methods

### Ethics statement

This study was carried out in accordance with the principles of the Basel Declaration and recommendations of Universidad Austral de Chile committee. The protocol was approved by the biosafety institution of the Universidad Austral de Chile (No 008/17). The samplings were carried out according to the authorisation granted by the Subsecretaria de Pesca y Acuicultura (SUBPESCA, Rex No. 2898/2015). The animals involved in this study were minor invertebrates (Mollusca: Bivalves: Mytilidae).

### Mussel collection

**Fig 1:**
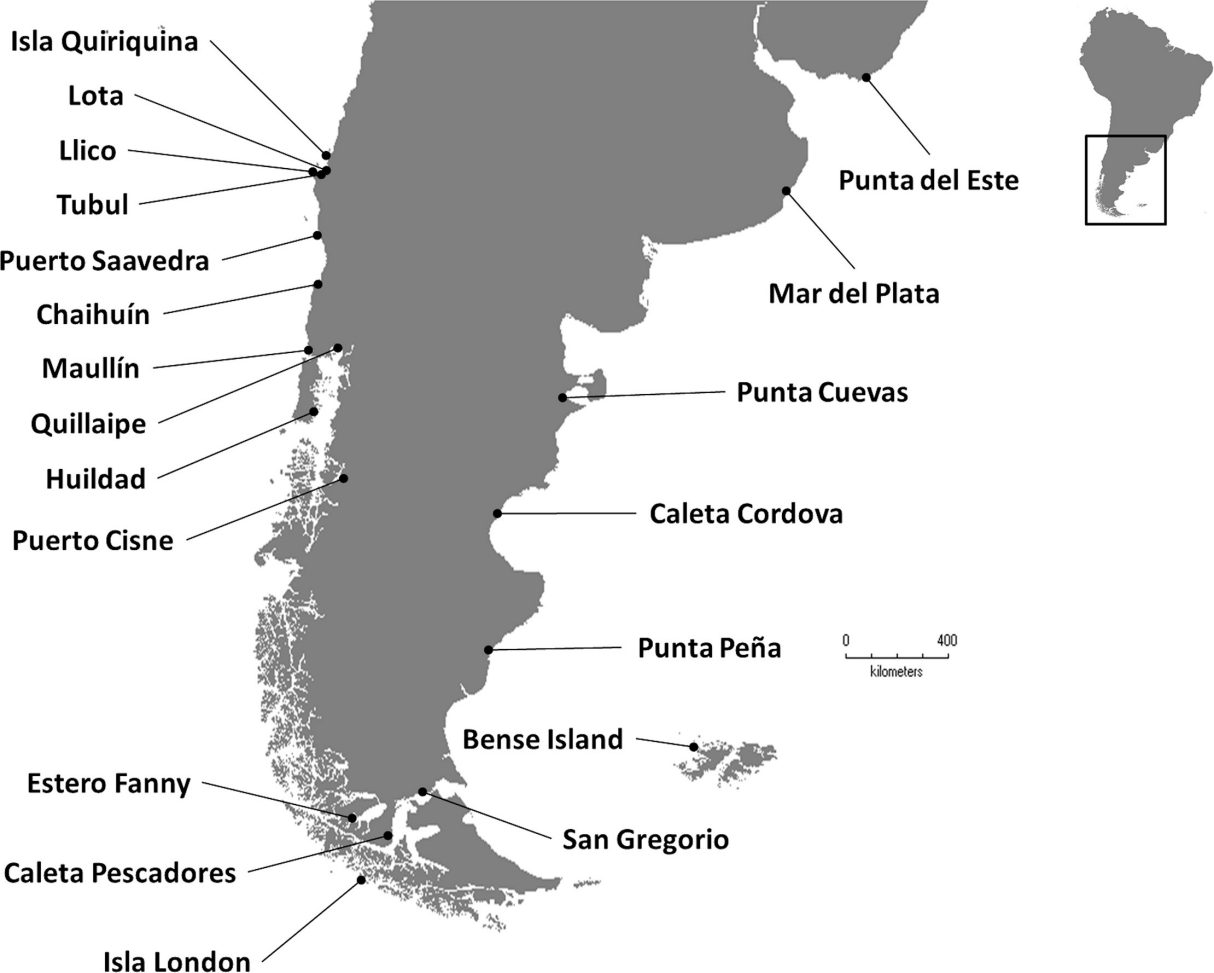
Map of the collection sites of smooth-shelled blue mussels (*Mytilus* spp.) in South America, including Chile, Argentina, the Falkland Islands and Uruguay.

Mussels were collected from the intertidal region by hand or from the shallow subtidal zone by SCUBA divers. Samples (8 to 20 mussels per site, mean = 17.4) were collected between 2002 and 2016 at 20 sites in Chile, four in Argentina, one in Uruguay and one from the Falkland Islands (Fig 1; Table 1). In total, 348 mussels were sampled within a size range of 27.3 to 65.9 mm shell length. In addition, reference samples (mantle tissue) from the Northern hemisphere including Canada (Bras d’ Or lake), UK (Cornwall) and Spain (Moaña-Pontevedra) were used.

**Table 1:** Collection site information in Argentina, Chile, the Falkland Islands and Uruguay, including habitat (subtidal or intertidal), geographical coordinates, number of mussels (*Mytilus* spp.) collected (N), and date of collection (DD.MM.YYYY).

Site	Habitat	Coordinates	N	Date
**ARGENTINA**				
Mar del Plata	S	38°00’32.0’’S; 57°31’48.1’’W	10	02.08.2010
Punta Cuevas	S	42°46’47.0’’S; 65°00’06.0’’W	10	15.07.2014
Caleta Cordova	S	45°45’64.0’’S; 67°21’40.3’’W	20	22.07.2015
Punta Peña	S	49°14’45.0’’S; 67°40’18.0’’W	20	05.09.2010
**CHILE**				
Isla Quiriquinas	I	36°37’49.9’’S; 73°03´09.2’’W	20	21.01.2016
Lota	I	37°04’27.1’’S; 73°09´53.4’’W	20	26.01.2016
Tubul	S	37°13’37.3’’S; 73°26’02.2’’W	20	05.12.2015
Llico	S	37°10’06.9’’S; 73°33’41.5’’W	20	05.12.2015
Puerto Saavedra	S	38°46’44.7’’S; 73°24´32.3’’W	20	15.12.2015
Chaihuín	S	39°56’40.4’’S; 73°34´40.4’’W	20	22.11.2015
Maullín	I	41°37’25.3’’S; 73°35´36.9’’W	20	27.11.2015
Quillaipe	I	41°32’59.5’’S; 72°45´14.0’’W	20	16.12.2015
Huildad	I	43°03’02.8’’S; 73°34´21.1’’W	20	28.11.2015
Puerto Cisne	S	44°44’11.4’’S; 72°41´07.9’’W	20	22.02.2016
San Gregorio	I	52°34’01.8’’S; 70°04’13.8’’W	10	22.10.2013
Caleta Pescadores	I	53°21’06.2’’S; 70°57’27,8’’W	20	21.10.2013
Estero Fanny	S	53°05’04.6’’S; 72°18’39.6’’W	20	17.10.2013
Isla London	S	54°40’57.6’’S; 71°55’29.7’’W	10	15.09.2013
**URUGUAY**				
Punta del Este	I	34°58’05.8’’S; 54°56’48.4’’W	20	05.06.2014
**FALKLAND ISLANDS**				
Bense Island	S	51°28’59.9’’S; 60°30’00.1’’W	8	14.10.2002
Total number of mussels			348	

### DNA extraction and visualisation

Each mussel was dissected and a small piece of mantle edge tissue was fixed in 95% ethanol and stored at 4°C. A subsample of ~ 30 mg of mantle edge tissue from each individual was used for total genomic DNA extraction using a DNA kit according to the manufacturer’s instructions (Geneaid^®^). Sizes of amplified fragments were estimated from a 50 bp DNA ladder (Invitrogen^TM^) on gels stained with SYBR® Safe DNA.

### Molecular markers, amplification and alignment

Molecular markers consisted of one nuclear ribosomal gene, 18S rRNA, and two mitochondrial markers, 16S rRNA and cytochrome *c* oxidase subunit I (COI). Fragments of the COI, 16S and 18S genes were amplified using the universal primers LCO1490/HCO2198 [63], 16SAR/BR [64] and 1822F/F22 [65].

Standard PCR amplifications were carried out in 25 μL reaction mixtures containing 2 μL DNA template, 0.2 mM total dNTP, 2mM MgCl_2_, 0.4 mM each primer, 1U of Taq (Invitrogen^TM^), the manufacturer’s PCR buffer, and sterile distilled water. The PCR conditions involved an initial denaturation at 94°C for 5 min, followed by 30 cycles of 94°C for 30 s, annealing at 55°C for 30s and elongation at 72°C for 1 min, followed by further elongation at 72°C for 5 min.

*Mytilus* F-type mtDNA COI and 16S haplotypes are known to possess a more informative phylogenetic signal than M-type mtDNA haplotypes [18,47,66,67]. Because of this, only female mitochondrial sequences were considered in the present study. Amplicons were purified and sequenced by Macrogen (South Korea). Both sequence directions were determined, using the individual primers from the original reaction. DNA sequence was edited using Geneious® 11.0.4. (Biomatters Ltd). All new sequences have been deposited in GenBank under accession numbers MZ313196-3204; MZ313212-21; MZ313464-68; MZ356925-7040 (Table 2). On the other hand, mytilids sequences used in previous publications were downloaded from GenBank (Table 2). All nucleotide sequences were aligned using MAFFT v.7 [68] under the iterative method of global pairwise alignment [69], and default settings were chosen for all parameters.

**Table 2:** GenBank accession numbers and sample locations of sequences used for this study for COI, 16S and 18S DNA sequence variation (for Figs 2 and 3).

Species	Sample location	GenBank Accession Number	Reference
**COI**			
*Mytilus trossulus*	North Atlantic	AF242027-29	[70]
*M*. *trossulus*	North Atlantic	AF242033-35	[70]
*M*. *trossulus*	Damariscotta, Maine, USA	AF242031	[70]
*M*. *trossulus*	Mahome Bay, Canada	AY130061-63	[71]
*M*. *trossulus*	Penn Cove, USA	AY130064/66/67	[71]
*M*. *trossulus*	Bras d’Or lake, Canada	MZ313466	This study
*Mytilus edulis*	Tjárno, Sweden	AY723898	[71]
*M*. *edulis*	Tjárno, Sweden	AY723900	[71]
*M*. *edulis*	Tjárno, Sweden	AY723912/13	[71]
*M*. *edulis*	Hanko, Finland	AY130046	[71]
*M*. *edulis*	Beaufort, USA	AF241937	[70]
*M*. *edulis*	Antigonish, Canada	AF241951	[70]
*M*. *edulis*	Cornwall, UK	MZ313468	This study
*M*. *edulis*	Punta Cueva, Argentina	MZ313464	This study
*M*. *galloprovincialis*	Samos, Greece	AY130054/60	[71]
*M*. *galloprovincialis*	Chioggia, Italy	AM905222	[18]
*M*. *galloprovincialis*	N/A	AF242015	[70]
*M*. *galloprovincialis*	Dichato, Chile	AM905177-79	[18]
*M*. *galloprovincialis*	Nedlands, Australia	AM905214	[18]
*M*. *galloprovincialis*	Paternoster, South Africa	AM905217	[18]
*M*. *galloprovincialis*	Green Cape, Australia	HQ864842	[67]
*M*. *galloprovincialis*	Tura Head, Australia	HQ864854	[67]
*M*. *galloprovincialis*	Moaña-Pontevedra, Spain	MZ313467	This study
*Mytilus* sp	Dunedin, NZ	AM905146-48	[18]
*Mytilus sp*	Wellington, NZ	AM905154	[18]
*Mytilus sp*	Cloudy Bay, Tasmania	AM905161/62	[18]
*Mytilus sp*	Simpson’s Bay, Tasmania	AM905166	[18]
*Mytilus sp*	Hobart, Tasmania	AM905170	[18]
*Mytilus sp*	Kerguelen Island	AM905201	[18]
*Mytilus sp*	Snug, Tasmania	HQ864836	[67]
*Mytilus sp*	Tura Head, Australia	HQ864848	[67]
*Mytilus sp*	Green Cape, Australia	HQ891001	[67]
*Mytilus sp*	Auckland Islands, NZ	MZ313465	This study
*M*. *californianus*	Monterey Bay, USA	MCU68776	[72]
*M*. *coruscus*	Zhoushan, East China Sea	KC139309-11	[73]
*Choromytilus chorus*	Colún, Chile	MT103131	This study
*Choromytilus chorus*	Concepción, Chile	JF301720-23	Unpublished
**16S**			
*M*. *trossulus*	Oregon, USA	U22879	[47]
*M*. *trossulus*	Bras d’Or lake, Canada	MZ313216	This study
*M*. *edulis*	Whitsand Bay, UK	AF023582	[74]
*M*. *edulis*	Delaware, USA	AF023550	[74]
*M*. *edulis*	Cornwall, UK	MZ313217	This study
*M*. *edulis*	Punta Cueva, Argentina	MZ313215	This study
*M*. *galloprovincialis*	Paternoster, South Africa	AM904597	[18]
*M*. *galloprovincialis*	Lota, Chile	MZ313219	This study
*M*. *galloprovincialis*	Moaña-Pontevedra, Spain	MZ313221	This study
*M*. *chilensis*	Puerto Cisne, Chile	MZ313213	This study
*M*. *chilensis*	Quillaipe, Chile	MZ313212	This study
*M*. *chilensis*	Maullin, Chile	MZ313218	This study
*M*. *chilensis*	Punta del Este, Uruguay	MZ313214	This study
*Mytilus sp*.	Auckland Islands, NZ	MZ313220	This study
*M*. *californianus*	San Diego, California, USA	AF023600	[74]
*M*. *coruscus*	China	AF317545	Unpublished
*Choromytilus chorus*	Concepción, Chile	EU636213	Unpublished
**18S**			
*M*. *trossulus*	Not registered	L24490	[75]
*M*. *trossulus*	Bras d’Or lake, Canada	MZ313196	This study
*M*. *edulis*	Helgoland, Gemany	AY527062	[76]
*M*. *edulis*	Cornwell, UK	MZ313197	This study
*M*. *galloprovincialis*	South Africa	DQ640507	[51]
*M*. *galloprovincialis*	Lota, Chile	MZ313198	This study
*M*. *galloprovincialis*	Moaña-Pontevedra, Spain	MZ313203	This study
*M*. *chilensis*	Puerto Cisne, Chile	MZ313199	This study
*M*. *chilensis*	Quillaipe, Chile	MZ313201	This study
*M*. *chilensis*	Maullin, Chile	MZ313200	This study
*M*. *chilensis*	Punta del Este, Uruguay	MZ313202	This study
*Mytilus sp*.	Auckland Islands, NZ	MZ313204	This study
*M*. *californianus*	Freshwater Bay, USA	L33449	[77]
*M*. *coruscus*	Fuding, China	EF613242	[78]
*Ch*. *chorus*	Chile	DQ640540	[51]

N/A–site location of collection not provided.

### Phylogenetic analyses

**Fig 2:**
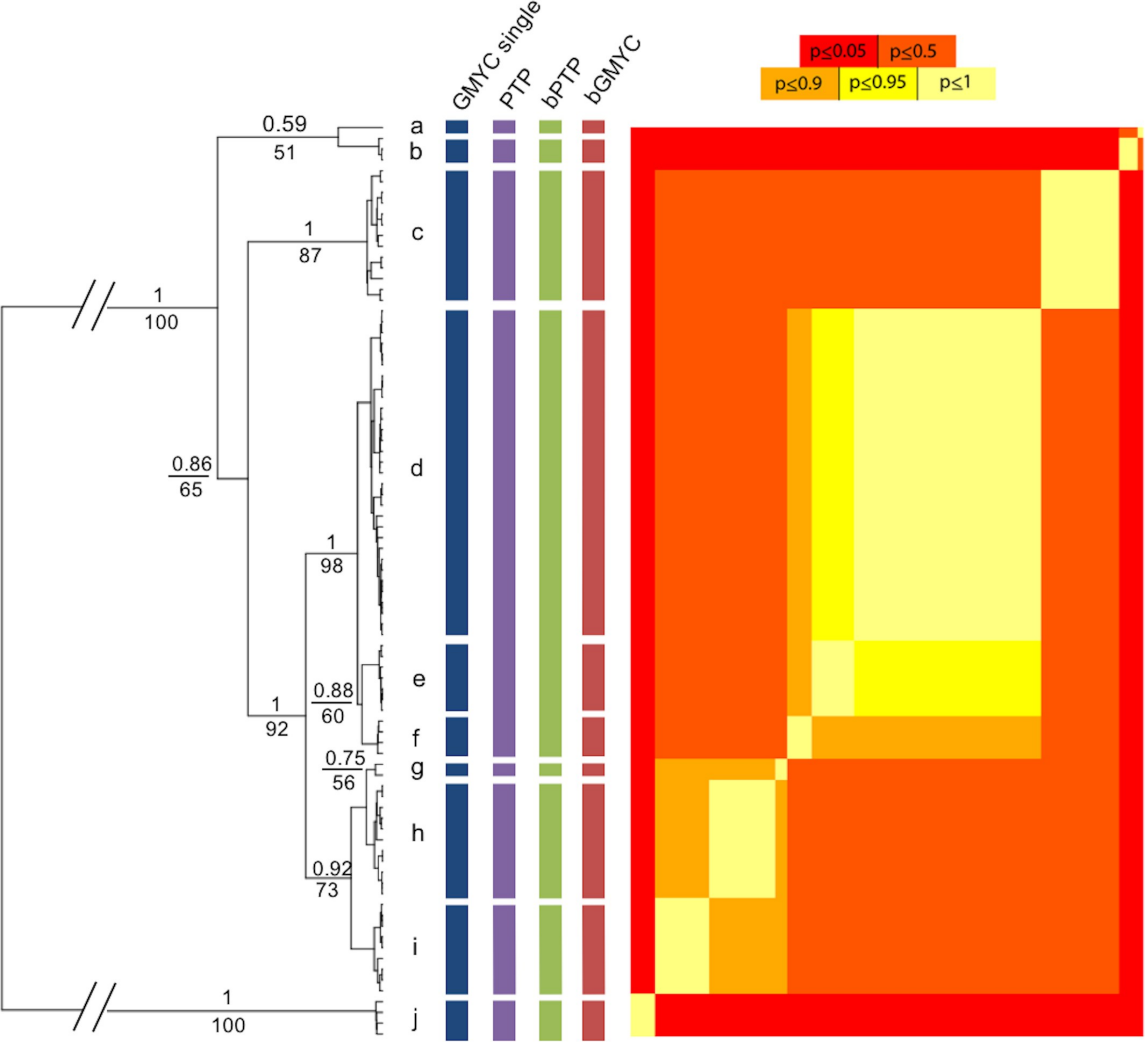
**To the left is the maximum clade credibility tree from the Bayesian analyses based on cytochrome oxidase subunit 1 (COI) sequences with results of the single threshold GMYC model (blue vertical lines), bGMYC (red vertical lines), PTP (purple vertical lines) and bPTP (green vertical lines).** Values above tree branches are Bayesian posterior probabilities/maximum likelihood bootstrap values. To the right the heat-map represents a sequence-by-sequence matrix where cells are coloured by the posterior probability that the corresponding sequences are conspecific, allowing for the visualisation of uncertainty in species limits returned by the bGMYC method. The letters correspond to clades a = *Mytilus californianus* from USA (Monterrey Bay); b = *Mytilus coruscus* from China (Zhouhan); c = *Mytilus trossulus* from USA (Damariscotta and Penn Cove) and Canada (Mahome Bay and Bras d’Or Lake); d = *Mytilus chilensis* from Chile (Maullín, Quillaipe Puerto Saavedra, Caleta Pescadores, Huildad, Estero Fanny, Isla London, Chaihuín, San Gregorio and Puerto Cisne), Argentina (Mar del Plata and Punta Peña), Uruguay (Punta del Este) and Kerguelen island; e = *Mytilus* spp. from Australia (Tura Head and Green Cape) and Tasmania (Cloudy Bay, Hobart, Simpson’s Bay and Snug); f = *Mytilus* spp. from New Zealand (Auckland Islands, Dunedin and Wellington), g = *Mytilus* spp from N/A and Finland (Hanko); h = *Mytilus galloprovincialis* from Italy (Chioggia), Greece (Samos), Australia (Green Cape, Nedlands, Tura Head), South Africa (Paternoster), Chile (Dichato, Lota); i = *Mytilus edulis* from Sweden (Tjárno), USA (Beaufort), Canada (Antigonish), Argentina (Punta Cueva) and the Falkland Islands (Bense Island); j = outgroup *Choromytilus chorus* from Chile (Colún).

**Fig 3:**
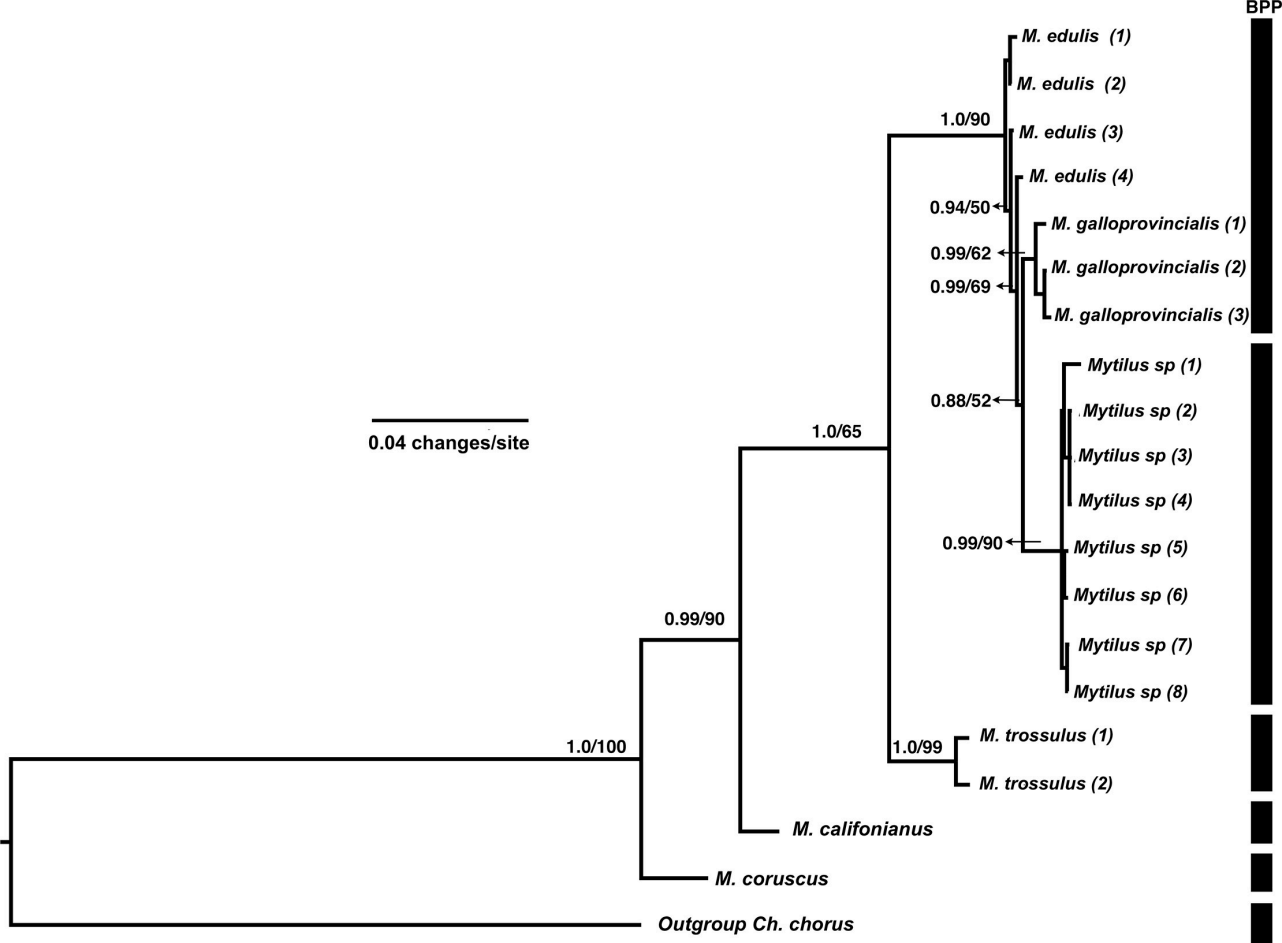
Bayesian tree using the concatenated matrix data set (COI+16S+18S). The numbers above branches represent the posterior probability/bootstrap support values. Bar on the right of the tree indicate the species limits as proposed by BPP (multilocus analysis). *M*. *edulis* = (1) Canada, (2) Sweden, (3) Cornwall, UK, (4) Punta Cueva, Argentina; *M*. *galloprovincialis* = (1) Moaña-Pontevedra, Spain, (2) Lota, Chile, (3) South Africa; *Mytilus* sp. clade = (1) Auckland Islands, New Zealand, (2–3) Quillaipe, Chile, (4) Maullin, Chile, (5) Punta del Este, Uruguay, (6) Maullín, Chile, (7–8) Puerto Cisne, Chile; *M*. *trossulus* clade = (1) Penn Cove, USA, (2) Bras d’Or lake, Canada. Sequence details are provided in Table 2.

Phylogenetic trees were constructed using Maximum Likelihood (ML) and Bayesian inference (BI): (i) with species of the family Mytilidae (S1 Fig) and (ii) with species of the genus *Mytilus*. Evolutionary models and partitioning strategies were evaluated with PartitionFinder v2.1.1 [79], which identified the best partition using the Bayesian Information Criterion, BIC [80]. A ML tree was inferred using GARLI v2.0 [81] with branch support being estimated by nonparametric bootstrap (BS) (200 replicates). Bayesian analyses were performed using MrBayes v3.2 [82]. Each Markov chain was started from a random tree and run for 5.0x10^7^ generations with every 1000^th^ generation sampled from the chain. Stationarity was checked as suggested in Nylander et al. [83]. All sample points prior to reaching the plateau phase were discarded as “burn-in”, and the remaining trees were combined to find the *a posteriori* probability of phylogeny. Analyses were repeated four times to confirm that they all converged on the same results. Posterior probability (PP) values >0.90 were taken as statistical support for a clade being present on the true tree [84]. The support values (PP and BS) were located in each node (Figs 2 and 3).

### Species delimitation within the *Mytilus* species complex

We employed five different methods for species delimitation, including four separate single locus (COI) analyses and a combined multilocus (COI + 16S + 18S) approach. We recognise that these sorts of models are only as good as the data that are used to test them, and for this reason we are interested to determine if a single marker or a multilocus approach is most powerful.

Firstly, we used the Generalized Mixed Yule Coalescent model (GMYC single), a method specifically developed for only one mitochondrial locus [5], and for when the majority of phylogenetic signal is found in mtDNA. This algorithm estimates the number of ‘‘species” by classifying the branching rates of a phylogram as being the result of interspecific or intraspecific lineage branching patterns (*sensu* Pons et al. [5]). After removing duplicate sequences (COI) because they may cause problems with downstream GMYC analyses [85], the best-fitting substitution model was chosen with the help of PartitionFinder v2.1.1 [79]. Using unique haplotypes, we built an ultrametric phylogenetic tree (Fig 2) in BEAST v1.8.1 [86]. We ran phylogenetic analysis under a lognormal relaxed clock set to an evolutionary rate of 9.51 x 10^−8^ [70] considering a coalescent tree with constant population size, using a random starting tree, and with 1 x 10^8^ Markov Chain Monte Carlo (MCMC) generations sampled every 1,000^th^ generation. We implemented two independent runs and combined results using LogCombiner v1.8.1 [86], burning the first 25% of the samples and then using Tracer v1.5 [87] to check for minimum adequate Effective Sample Size (ESS values > 200) and to visually inspect stationarity and convergence by plotting likelihood values. A consensus was built with TreeAnnotator 1.8.1 [86] using the maximum clade credibility method. This tree was used as input to estimate GMYC single in the package SPLITS (SPecies LImits by Threshold Statistics—[88]) using R v3.0.1 [89].

Secondly, we used a Bayesian version of this model (bGMYC), which addresses the uncertainty in the trees by sampling over a posterior distribution of sampled trees [90]. bGMYC analyses were performed by running the eponymous R package on the 100 trees sampled during the MCMC in BEAST v1.8.1 (we discarded the first 90% trees as ‘burn-in’). We ran each tree for 50,000 generations, discarding the first 40,000 generations as burn-in and using thinning intervals of 100 generations (as recommended by the authors). The threshold parameter priors (t1 and t2) were set at 2 and 96, and the starting parameter value was set at 25. In the case of bGMYC analyses, the convergence of the MCMC was assessed by checking the evolution graph of the posterior probability against the number of generations, as advised in the bGMYC tutorial.

Thirdly, the Poisson Tree Processes (PTP) model [8] was employed to infer molecular clades based on our inferred molecular phylogeny. The PTP method estimates the mean expected number of substitutions per site between two branching events using the branch length information of a phylogeny and then implements two independent classes of Poisson processes (intra and inter-specific branching) before clustering the phylogenetic tree according to the results (*sensu* Zhang et al. [8]).

Fourthly, we used bPTP, which is an updated version of the original maximum likelihood PTP (maximum likelihood PTP search result is part of the bPTP results). It adds Bayesian support (BYS) values to delimited species on the input tree. A higher BYS value on a node indicates all descendants from this node are more likely to be from one species. The two analyses (PTP and bPTP) were conducted on the web server for PTP (available at http://species.h-its.org/ptp/) using the MrBayes topology as advocated for this method [8,91].

Fifthly, we used the program BPP [10] to compare different models of species delimitation and species phylogeny in a Bayesian framework, accounting for incomplete lineage sorting due to ancestral polymorphism and gene tree versus species tree conflicts [6,92]. This program conducts multilocus, coalescent-based analyses requiring a guide tree and specification of two priors involving population size and divergence time. Thus, we used the A10 mode, which delimits species using a species tree estimated in BEAST v2.4.8 [93,94]. The population size parameters (θs) were assigned the gamma prior G (9, 100). The divergence time at the root of the species tree (τ0) was assigned the gamma prior G (8, 1000), whilst the other divergence time parameters were assigned the Dirichlet prior [6] (Eq 2). Each analysis was run at least twice to confirm consistency between runs.

The taxonomic index of congruence (Ctax) between pairs of species delimitation methods was estimated, following Miralles and Vences [95]. To identify the most congruent species delimitation approaches, the mean Ctax value for each method was also estimated (S1 Table).

### Analysis of broad spatial scale genetic structure

To test the robustness of the species delimitation approach we employed an independent analytical approach to identify population clusters of blue mussels in South America. In principle, if the DNA-based species delimitation approach is accurate, then when applied to the population genetic structure of mussels this should be reflected by different spatially explicit clusters of mussels in South America, for example on the Atlantic and Pacific coasts. This methodology also has the benefit of being able to identify the occurrence and location of regions of interbreeding between two (or more) different clusters of mussels. In addition, the presence of non-native species, such as Northern hemisphere *M*. *galloprovincialis*, may be identified. The spatially explicit Bayesian clustering program Geneland 3.2.4 [96] (an extension of program R 3.1.2. R Development Core Team) was used to investigate spatial genetic structure using COI data (Table 1). We converted variable base sites into bi-allelic (allele-like) data, so that the COI input file was a binary file. We ran ten independent runs, where the parameters for possible populations were K = 1–18, and the number of MCMC iterations was 4,000,000, saving every 100 steps (S2 Fig). After comparing the results of the analyses, we selected the run with the highest posterior probability and post-processed it for graphical display. A burn-in of 10,000 generations (20%) was trimmed from the posterior in the post-processing. A contour map of the posterior mode of population membership was drawn to visualise genetic substructure within South America.

## Results

### Phylogenetic relationships within the genus *Mytilus*

Two alignments were performed. First, we aligned the COI data for a total of 600 sites. Testing for the best fit substitution model resulted in the selection of models GRT+I+G (1^st^, best fit), TVM+G (2^nd^) and TIM+G (3^rd^) for COI codon position (Fig 2). Then we aligned the three DNA markers for a total of 2638 sites: 441 were variable and 206 were phylogenetically informative. Two of three markers corresponded to mitochondrial dataset with a total of 1068 nucleotide sites, of which 339 were variable and 186 were phylogenetically informative. The evolutionary models and the partitioning strategy obtained in Partitionfinder were SYM+I (1^st^), F81 (2^nd^), HKY+I (3^rd^) (COI), HKY+G (16S) and K80+I (18S).

Known phylogenetic relationships of species within the genus *Mytilus* based on COI sequence variation were generally well captured. *Mytilus californianus* Conrad, 1837 (Fig 2, group a) and *Mytilus coruscus* Gould, 1861 (Fig 2, group b) formed a divergent clade from the other species of *Mytilus*, but with relatively low support (BS = 51%, PP = 0.59). *M*. *trossulus* (Fig 2, group c), *M*. *edulis* (Fig 2, group i) and *M*. *galloprovincialis* (Fig 2, group h) all formed reasonably well-supported clades, with *M*. *edulis* as the sister species of *M galloprovincialis*. Mussels from Chile (Fig 2, group d) formed a well-supported clade, different from the species of the Northern hemisphere. The *M*. *chilensis* clade appeared to be monophyletic with respect to native *Mytilus* spp. from Australia (Fig 2, group e) and New Zealand (Fig 2, group f), although with low bootstrap support and posterior probability support (BS = 60%, PP = 0.88).

The placement of samples within the clades from our different geographic collecting sites was generally consistent with expectations, based on published data. The *M*. *chilensis* clade (presumptive native mussel diversity on the Pacific coast of South America) contained native mussels from Chile, but also included mussels from the Atlantic coast of Argentina and Uruguay, as well as from the Indian Ocean location of the Kerguelen Islands. The *M*. *galloprovincialis* clade contained mussels from the two Mediterranean Sea sites (Italy and Greece), but also contained mussels from Australia, South Africa and Chile. The *M*. *edulis* clade contained mussels from Sweden and the Atlantic coasts of the USA and Canada, but also included mussels from Argentina and the Falkland Islands (Fig 2). A final group, of mixed geographic origin, was also recognised, containing mussels from the Atlantic coast of the USA and Finland (Baltic Sea) (Fig 2; AY130046 and AF242015, see detail in Table 2). This geographically anomalous group was described as clade "g". However, this clade showed low support.

### Species delimitation analyses

The most congruent result amongst single- and multi-locus analyses recognised eight monophyletic lineages (including the outgroup) as different species (Fig 2 - PTP and bPTP; mean Ctax = 0.89, see all Ctax values in S1 Table). The *Mytilus chilensis* and *M*. *trossulus* lineages were recovered in phylogenetic analyses and were also supported in the Bayesian tree of concatenated sequences (Figs 2 and 3; BS >90; PP>0.99).

The total GMYC analysis, including the outgroup, identified ten entities or putative species (CI = 5–29). The bGMYC analysis identified ten species with a posterior probability >0.95 and nine species with posterior probability >0.90 where clades ’d’ and ’e’ were grouped together (Fig 2), whereas the PTP (with a speciation rate = 31.830; coalescent rate = 261.281; null logl = 308.567; max logl = 341.371; *P*-value < 0.001) and the bPTP analyses both identified eight species (Fig 2).

In the bGMYC the distribution of ratios of the Coalescence to Yule rates was above 0, and without negative values (S3 Fig), indicating that the model is a good approximation of the reality of the data. Bayesian GMYC analyses detected *Mytilus californianus*, *M*. *coruscus*, *M*. *trossulus*, *M*. *galloprovincialis* and *M*. *edulis* in the Northern hemisphere. Three species were detected in the monophyletic clade (PP = 1; BS = 98%) of the native mussels of the Southern hemisphere (d = *Mytilus chilensis* from Chile, Argentina, Uruguay and Kerguelen island; e = *Mytilus* sp. from mainland Australia and Tasmania; and f = *Mytilus* sp. from NZ–Fig 2). The results from the PTP, bPTP, GMYC and bGMYC analyses differ in their ability to differentiate between species in Australia versus New Zealand. However, the difference in Ctax values between PTP and bGMYC was marginal (PTP mean Ctax = 0.89 and bGMYC mean Ctax = 0.82, see S1 Table), indicating that this clade would include three species (d, e and f—Fig 2).

Results of the BPP species delimitation analyses are shown in Fig 3. In this multilocus analysis five putative *Mytilus* species were identified, with a speciation rate = 43.480; coalescent rate = 869.620; null logl = 717.926; max logl = 807.662; and *P*-value < 0.001. As expected, both *M*. *coruscus* and *M*. *californianus* were recognised as species. *M*. *trossulus* (from Canada and USA) was also recognised as a distinct species. Although there was clear separation of *M*. *edulis* (containing native mussels from Canada, Sweden, the UK and Argentina) from *M*. *galloprovincialis* (containing native mussels from Spain plus introduced mussels from Chile and South Africa) within the tree, the BPP did not recognise these two groups as separate species. The fifth and final group recognised by the BPP was a mixed Southern hemisphere (putatively *M*. *chilensis*) clade, containing native mussels from the Auckland Islands (New Zealand Southern Ocean), Chile and Uruguay.

#### Analysis of broad spatial scale genetic structure

**Fig 4:**
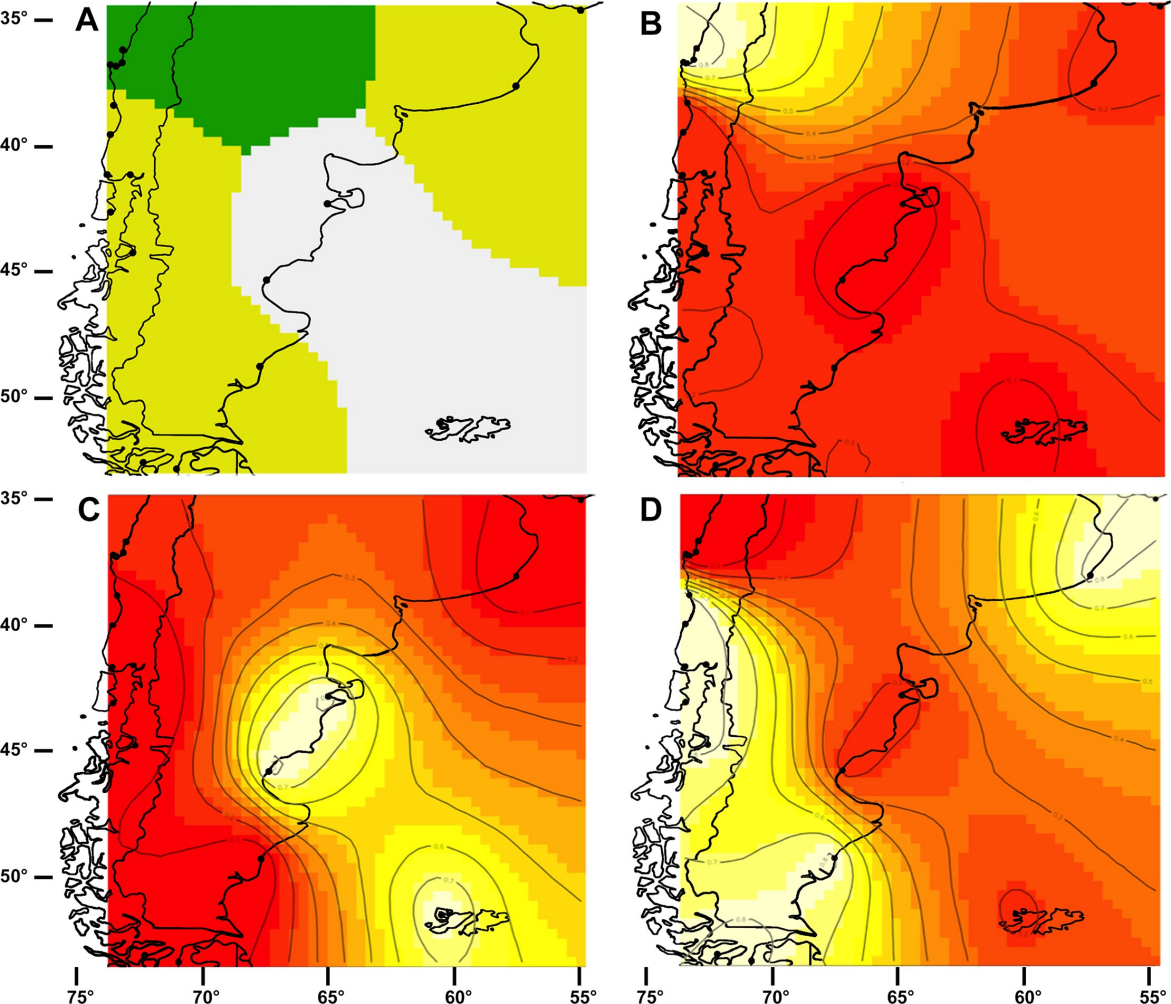
Geneland results for *K* = 3 groups using the spatial model with correlated allele frequencies (based on COI). (A) Map of estimated posterior probability of population membership (by posterior mode)–different colours represent distinct genetic groups; plots representing the assignment of pixels to: B) introduced *Mytilus galloprovincialis* in Chile, (C) mussels from the Atlantic coast of South America, and (D) *M*. *chilensis* mussels from the Pacific and southern Atlantic coasts of South America. The highest membership values are in white and light yellow and the contour lines indicate the spatial position of genetic discontinuities between populations.

Based on COI sequence variation, the software program Geneland, which takes into account spatial information, indicated that *K* = 3 groups was the most likely structure in South America (Fig 4A). The three groups were geographically clustered, with one group being centred around northern Chile (Northern hemisphere *M*. *galloprovincialis*—highlighted in yellow Fig 4B), the second group being centred around the coast of Argentina and the Falkland Islands (native southern Atlantic Ocean mussels–Fig 4C), and the distribution of the third group spanning southern Chile, the Straits of Magellan, and the southern part of Argentina (native *M*. *chilensis*–Fig 4D). This latter group also included the mussels from Uruguay. The assignment probabilities of individuals to their respective clusters were high, at ≥ 0.90 (Fig 4).

## Discussion

For the first time, species delimitation models have been applied to both single locus (COI) and multilocus (COI+16S+18S) datasets to examine in a robust, repeatable and objective manner the taxonomic relationships within the genus *Mytilus*. Our primary focus has been to examine the diversity of the genus *Mytilus* from South America, including the Falkland Islands. In this test case study, blue mussels collected from sites around South America have been examined in the context of the global diversity of the genus. In addition, genetic sequence variation (COI) that has been used in previous studies where they addressed the evolutionary history of the *Mytilus edulis* complex (i.e. [18,67,70,71]) was analysed for the South American mussels in a geospatial framework (Geneland).

### Species delimitation within the *Mytilus edulis* species complex

Our samples cover the distribution of *Mytilus* in South America, with the exception of Brazil where the introduction of *M*. *galloprovincialis* has recently been reported [97]. Although the most congruent species delimitation analysis (using COI) defined *M*. *coroscus*, *M*. *californianus*, *M*. *galloprovincialis*, *M edulis*, *M*. *trossulus*, and *M*. *chilensis*, it did not provide evidence for the existence of the species *Mytilus platensis* d’Orbigny, 1846 on the Atlantic coast (Argentina or Uruguay). At sites where *M*. *platensis* is expected, the species delimitation models (SDMs) identified the Northern hemisphere species, *Mytilus edulis*, consistent with several earlier interpretations of the affinity of *M*. *platensis* with Northern hemisphere *M*. *edulis* (e.g., McDonald et al [13], Borsa et al. [24]). On the other hand, our analyses corroborate the monophyly of native mussels on the Pacific coast of South America (*Mytilus chilensis* Hupé, 1854). In the Southern hemisphere, *M*. *chilensis*, along with two distinct evolutionary lineages from New Zealand (putative *M*. *aoteanus* Powell, 1958) and Australia (putative *M*. *planulatus* Lamarck, 1891) coexist with *Mytilus galloprovincialis* (the Mediterranean mussel), which has been introduced to parts of Chile (Bahía de Concepción), Brazil, Australia and New Zealand [18,20,22,35,41,67,97–100]. Although the most congruent unilocus analysis (PTP: mean Ctax = 0.89) delimited in one species the blue mussels native to Argentina, Chile, Uruguay, Kerguelen Islands, New Zealand, Australia and Tasmania (see Fig 2 - groups d, e, f), the recovery of species from the General Mixed Yule Coalescent model (mean Ctax = 0.82) separated this clade into three species (d = Chile, part of Argentina, Uruguay and Kerguelen Islands; e = Australia; and f = New Zealand). Many previous studies have reported pronounced genetic differentiation between mussels from Australia/Tasmania and New Zealand (e.g., Gérard et al. [18], Sanjuan et al. [101], Pickett et al. [102]). Therefore, the SDM results support the taxonomy described by Lamy [103] and Powell [104] who separated the Southwest Pacific Ocean mussels into two species: New Zealand animals were classified as *Mytilus aoteanus* Powell, 1958 (currently synonymous with *M*. *galloprovincialis*—WORMs), and those of Australia as *Mytilus planulatus* Lamarck, 1819.

Overall, the GMYC model can be considered suitable for the data. The bGMYC provides reliable results when the branching rate of the coalescent process is higher than the branching rate under a Yule process. For our data the distribution of the ratio of the coalescence rate to the Yule rate is between one and two, with no negative values (S3 Fig). Nevertheless, we recommend studying mussels native to Australia and New Zealand to test the specific hypothesis within the framework of species delimitation.

Generally, the analysis of broad spatial scale genetic structure based on the COI sequence variation used in the spatially explicit Geneland analysis supported the species delimitation models, and identified three main groups within South America. Invasive Northern hemisphere *M*. *galloprovincialis* was observed in northern Chile, in the vicinity of Concepcion, consistent with earlier reports of its occurrence here [20,35,105]. A second group of mussels was identified on the Atlantic Ocean coastline of Argentina and also in the Falkland Islands, corresponding to putative *M*. *platensis* (e.g., Zbawicka et al. [34]). However, according to the species delimitation analyses, these mussels correspond to clade i (*Mytilus edulis*—Fig 2). The third and largest group in terms of area of distribution was recorded for the southern Pacific Ocean coastline of South America, south of Punta Lavapié (37°20’), and included all of the Straits of Magellan and extended north onto the Atlantic Ocean coast of Argentina. This putative species is consistent with *M*. *chilensis* (e.g., Larraín et al. [43]). Interestingly, and perhaps somewhat surprisingly, the Uruguay population of Punta del Este, which is near the northern limit of *Mytilus* sp. on the Atlantic coast of South America, was also identified as belonging to this third group. We do not know if this population is a genuine member of the group or if perhaps it represents a localised introduction of putative *M*. *chilensis* into Uruguay. This clearly warrants further examination, but is beyond the scope of this study. The recent report of introduced Northern hemisphere *M*. *galloprovincialis* in southern Brazil [97] highlights how quickly the situation can change as records of invasive species establishment change from year to year.

### Advantages and limitations of the species delimitation approach

Species delimitation models have strengths and weaknesses and the outcome (interpretation of species-specific differences) is only as good as the data set being used (single versus multiple locus or marker; mitogenome versus nuclear genome; rapidly evolving versus conserved loci) and also will very much depend on the analytical approach (the model and its assumptions) employed.

The four different single locus (mtDNA) species delimitation tests identified nine (GMYC and bGMYC) or seven (PTP and bPTP) species, whereas the one multilocus (mtDNA + nDNA) test (BPP) identified only five species within the genus *Mytilus* (excluding the outgroup). GMYC [5] uses as input an ultrametric tree estimated from a single locus. The method models the transition point between cladogenesis and allele coalescence by assuming that the former will occur at a rate far lower than the latter. This results in a shift in the rate of branching of the genealogy that reflects the transition between species-level processes and population-level processes (taken from the review by Cartens et al. [2]). On the other hand, bGMYC takes into account phylogenetic uncertainty gene tree estimates using a Bayesian approach [90]. Both implementations of the GMYC are likely to delimit well-supported clades of haplotypes as independent lineages and as such may be prone to over delimitation (*sensu* Cartens et al. [2]). This probably explains the erroneous delimitation of clade g (see Fig 2).

The BPP method implements a reversible jump Markov chain Monte Carlo search of parameter space, which includes population divergence and estimated distributions of gene trees from multiple loci [6]. The method uses sequence data, and the user is asked to define the topology of the species tree [10]. Then, the posterior probability of the proposed nodes of the species tree is calculated. Whilst inaccurately specified guide trees can lead to false-positive delimitations, the accuracy of BPP is not dependent on its ability to estimate gene trees (*sensu* Cartens et al. [2]). Cartens et al. [2] indicated that the validation approaches (such as BPP) are often given more weight by empirical investigations because they explicitly model the process of lineage diversification. However, the results should always be interpreted with caution as the methods are not perfect. In our case, the validation of *M*. *chilensis* as the predominant species on the Pacific Ocean coast of South America is robust, whereas the validation of *M*. *platensis* on the Atlantic Ocean coast is not robust.

### Use of sequence data for the identification of species within the genus *Mytilus*

Many mitochondrial protein coding genes have been used to study phylogenetic relationships amongst species [106] due to the high rate of substitution occurring in the third codon position. However, the nuclear rDNA (e.g. 18S) is one of the most highly conserved DNA regions and has been used to reconstruct phylogenies from phyla to orders [107,108]. Whilst the joint use of nuclear and mitochondrial genetic data has been very useful for recovering a phylogeny in the Mytilidae [109], the 18S gene has a better phylogenetic signal in analysis between genders (e.g., Distel [65], Owada [110], Liu et al. [111]), whereas mitochondrial genes may have more utility in studies of species complexes (e.g. [112–115]). Because of this concatenation of DNA sequences from different genes (indeed, different genomes with very different properties including size, evolutionary rate, and mode of inheritance) with different strengths or depths of evolutionary signal, it is likely that the multilocus analysis (BPP) failed to recover the species of the Northern hemisphere (*M*. *edulis* and *M*. *galloprovincialis*), although they were evident in the unilocus analyses (PTP, bPTP and bGMYC). However, in both analyses of species delimitation the specific status of *Mytilus chilensis* is confirmed for animals that inhabit southern Chile, part of Argentina, Uruguay and the Kerguelen Islands.

### Comparison of species delimitation results with recent SNPs analyses

The species delimitation tests were performed on DNA sequence data, some of which has been used for decades to assess phylogenetic and phylogeographic variation (e.g. Wares and Cunningham [70], Riginos et al. [71]). The most recent analyses of Southern hemisphere *Mytilus* population genetic variation have employed highly variable nuclear DNA markers such as single nucleotide polymorphisms (SNPs). These markers have produced new insights into the taxonomy and phylogeography of native and introduced mussels from New Zealand [116], Chile [43], Argentina [34], Brazil [97] and Australia [100] and offshore islands [117]. Generally speaking the interpretation of the phylogeography, and therefore indirectly the taxonomy, of blue mussels from these Southern hemisphere regions has tended to be based on analyses such as STRUCTURE [118,119], AWclust [120], CA [121,122] and DAPC plots [123] that identify distinct genetic clusters, and by inference some degree of genetic isolation and therefore putative taxonomic identity. SNPs data sets usually provide the greatest level of detail (definition) of all genetic data sets used to date, but are not designed to work in a phylogenetics setting because their high mutation rates are generally not suited to such an approach. This is why taxonomic problems are usually approached from the phylogenetic and coalescence perspective. For the Southern hemisphere mussels, greatest congruence was observed between the GMYC single analysis of the COI data set and the SNPs-based phylogeographic and taxonomic interpretations [34,43,100,116,117]. Overall, the SNPs analyses indicate that in the Southern hemisphere there are clear genetic clusters, that is, distinct genetic entities, in the different major geographic areas surveyed: Chile (putative *M*. *chilensis*), Argentina and Uruguay (putative *M*. *platensis*), New Zealand (putative *M*. *aoteanus*), Australia (putative *M*. *planulatus*), plus individuals of mixed ancestry in offshore regions, such as the Falkland Islands (southern Atlantic Ocean), the Kerguelen Islands (southern Indian Ocean), and the Auckland/Campbell Islands of New Zealand (southern Pacific Ocean). The broad-scale phylogeography of Southern hemisphere blue mussels has recently been reviewed by Gardner et at. [124]: our SDM and Geneland results are consistent with interpretations presented by many authors that are described in this review. The congruence between the SNP and GMYC results is probably due to the fact that the precision of the GMYC model increases when a marker with a small effective population size and a high mutational rate is used [125]. Clearly, an important next step will be to test the species delimitation models on the SNPs data sets [34,43,116,117] to compare the objective results from the models against the subjective, and at times controversial, interpretation of the researchers in question in describing the taxonomy of Southern hemisphere blue mussels.

## Conclusions

One of the longstanding debates about the taxonomy of the *M*. *edulis* species complex, in particular in the Southern hemisphere, has revolved around some of the subtle differences that exist (for example, between native mussels from the Pacific Ocean coast of Chile versus the Atlantic Ocean coast of Argentina, or between South America and Australasia, or on remote offshore islands such as the Falkland Islands and the Kerguelen Islands), and whether these are minor differences that reflect an affinity to Northern hemisphere species (i.e., sub-species status), or larger evolutionary differences that may reflect distinct species. Ultimately, this interpretation may depend on the definition that is applied of what constitutes a biological species [4,126] or to what has been called individual researcher ‘ … taxonomic preconception.’ (Gérard et al. [18], p. 84). Regardless, the importance of reaching a standardised and universally agreed taxonomy is apparent, in particular in terms of food labelling, biosecurity and conservation [27,43,59]. The unusual basis of mtDNA inheritance in blue mussels, the close evolutionary relationships amongst the taxa, and the potential for hybridisation between any pair of co-occurring taxa makes the taxonomic classification and phylogenetic reconstruction for this group extremely challenging, using either morphological or molecular data [13–18,127]. It is these same challenges that make this group of mussels ideally suited for testing using species delimitation models, in particular with new markers such as SNPs. Whilst the application of five different species delimitation models in this case study focussed on South American mussels has not identified the full range of putative species identities, the approach nonetheless shows great promise if applied to more informative markers such as SNPs as well as to a larger data set with greater geographic coverage.

## Supporting information

S1 Fig Phylogenetic relationships of the Mytilidae (COI). Phylogenetic relationships of the Mytilidae reconstructed based on COI gene sequence variation using the ML and BI methods. Numbers above the branches are Bayesian posterior probabilities/likelihood bootstrap values (data set 123 sequences = 600 pb). Nucleotide model = (GTR+I+G)(TVM+G)(TIM+G). Access numbers in S2 Table.
(DOCX)

S2 Fig Geneland analysis. Number of clusters along the chain after burn-in.
(DOCX)

S3 Fig bGMYC analyses. The distribution of ratios of the coalescence rate to the Yule rate sampled in the analysis. If the values are above 0, without negative values, then the model is a good approximation of the reality of the data. In reference to Fig 2.
(DOCX)

S4 Fig Unrooted tree drawn from neighbour-joining analysis. From the sequences of Fig 4. A) introduced *Mytilus galloprovincialis* in Chile, B) *M*. *chilensis* mussels from the Pacific and southern Atlantic coasts of South America, C) mussels from the Atlantic coast of South America. Scale bar: Genetic distance.
(DOCX)

S1 Table Taxonomic index of congruence (Ctax). Taxonomic index of congruence (Ctax) calculated for each pair of approaches. Mean of all the Ctax values obtained involving a given approach (Mean Ctax) and total number of species supported by each approach (sp.) is indicated. Species delimitation approaches: General Mixed Yule Coalescent model (GMYC), Bayesian General Mixed Yule Coalescent model (bGMYC; 0.95), Poisson Tree Processes (PTP), bayesian Poisson Tree Processes (bPTP), Bayesian Species Delimitation (BPP).
(DOCX)

S2 Table GenBank accession numbers for S1 Fig. GenBank accession numbers of sequence used in the analysis of Fig 1S for gen cytochrome *c* oxidase subunit I (COI).
(DOCX)
