# Supplementary material for: Blue mussels of the Mytilus edulis species complex from South America: The application of species delimitation models to DNA sequence variation
Source: PLoS One. 2021 Sep 2;16(9):e0256961. doi: 10.1371/journal.pone.0256961 (PMC8412288; doi:10.1371/journal.pone.0256961)
Supplement: S1 Fig — Phylogenetic relationships of the Mytilidae reconstructed based on COI gene sequence variation using the ML and BI methods. Numbers above the branches are Bayesian posterior probabilities/likelihood bootstrap values (data set 123 sequences = 600 pb). Nucleotide model = (GTR+I+G)(TVM+G)(TIM+G). Access numbers in S2 Table. (DOCX) [file pone.0256961.s001.docx]

**
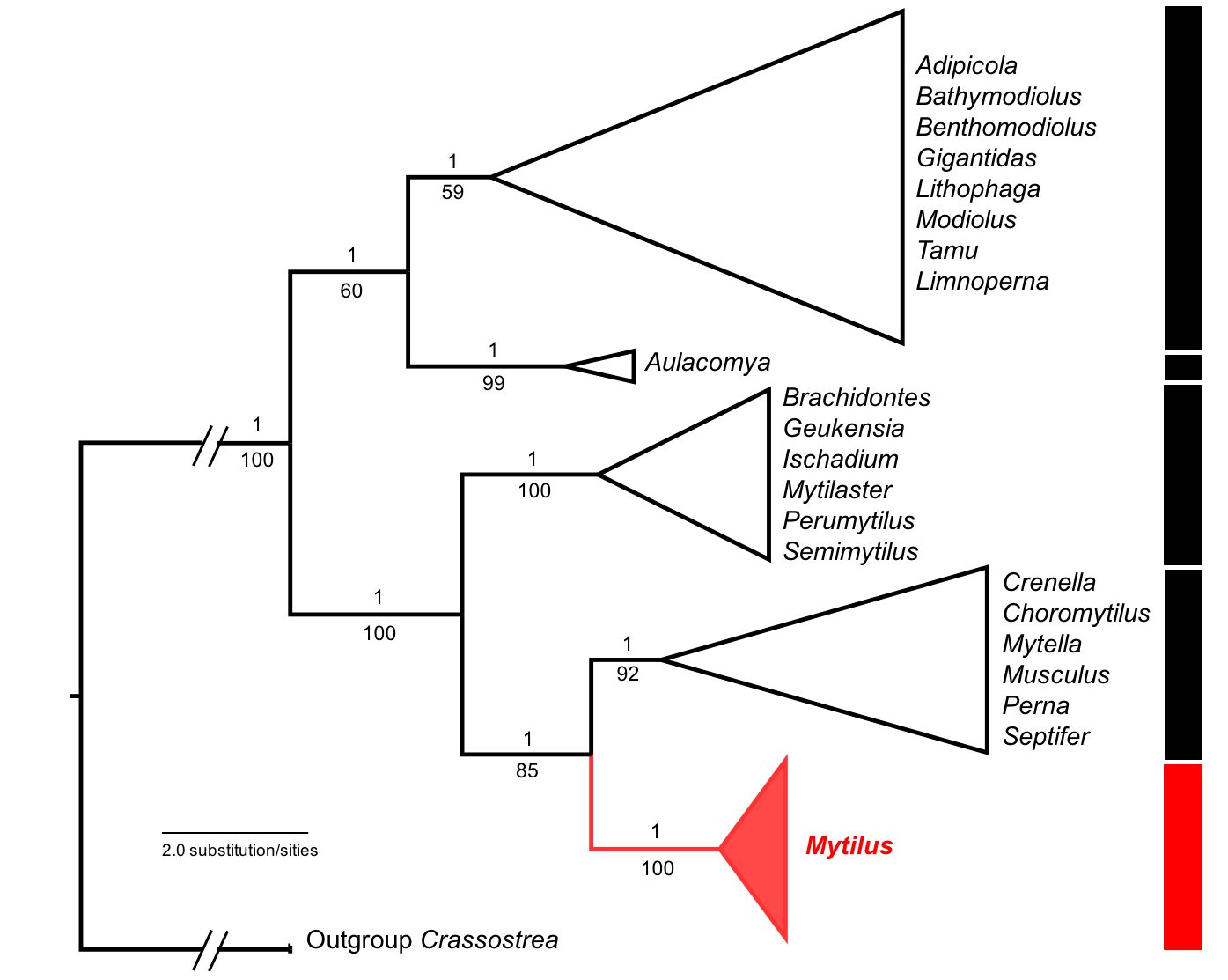
**

**S1 Fig.** **Phylogenetic relationships of the Mytilidae.** Phylogenetic relationships of the Mytilidae reconstructed based on COI gene sequence variation using the ML and BI methods. Numbers above the branches are Bayesian posterior probabilities/likelihood bootstrap values (data set 123 sequences = 600 pb). Nucleotide model = (GTR+I+G)(TVM+G)(TIM+G). Access numbers in Table S6.
