# Supplementary figures and images for: Blue mussels of the Mytilus edulis species complex from South America: The application of species delimitation models to DNA sequence variation
Source: PLoS One. 2021 Sep 2;16(9):e0256961. doi: 10.1371/journal.pone.0256961 (PMC8412288; doi:10.1371/journal.pone.0256961)

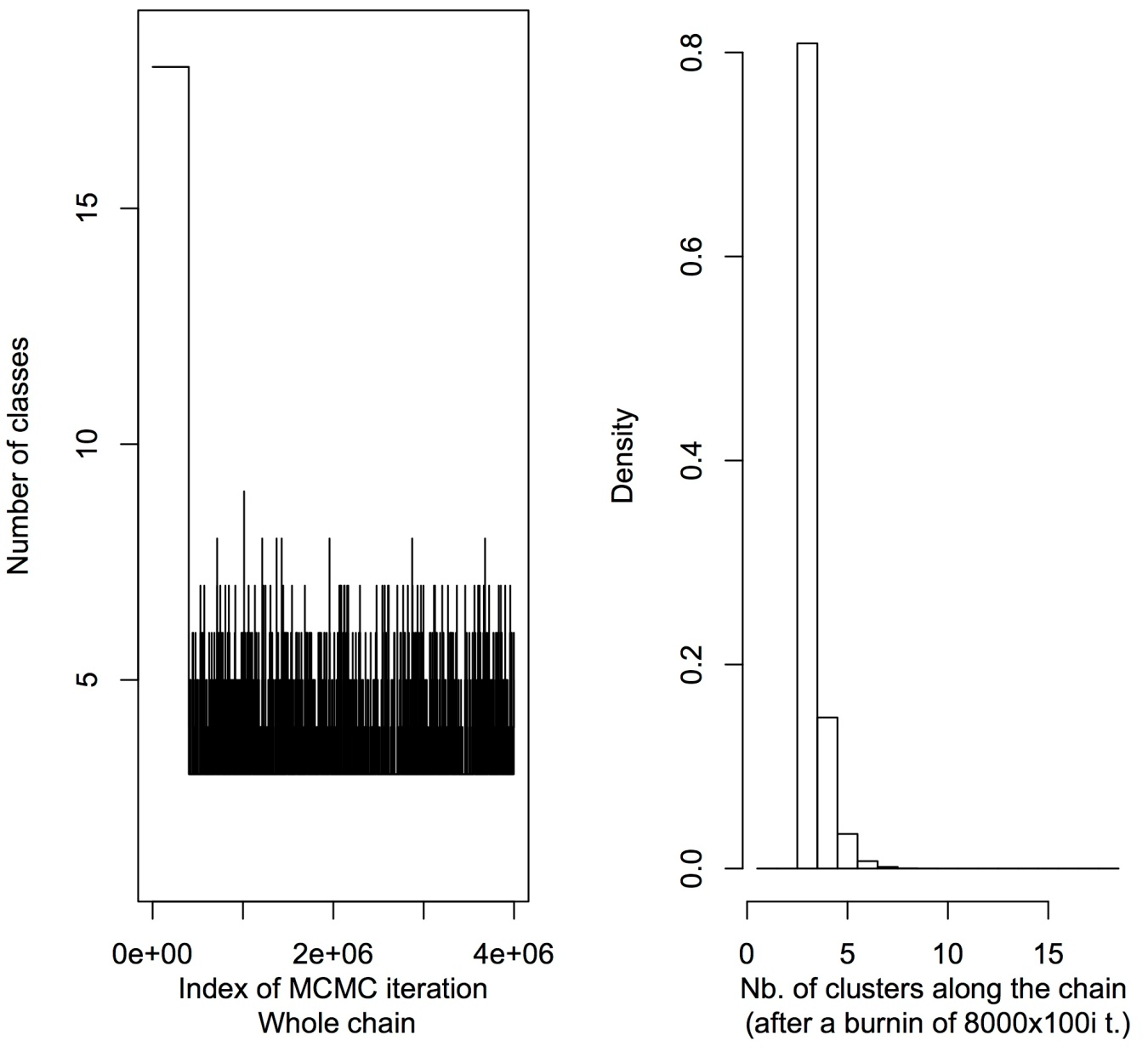


**S3 Fig.** **Geneland analysis**. Number of clusters along the chain after burn-in.

Supplement: S2 Fig — Number of clusters along the chain after burn-in. (DOCX) [file pone.0256961.s002.docx]
