# Supplementary material for: Blue mussels of the Mytilus edulis species complex from South America: The application of species delimitation models to DNA sequence variation
Source: PLoS One. 2021 Sep 2;16(9):e0256961. doi: 10.1371/journal.pone.0256961 (PMC8412288; doi:10.1371/journal.pone.0256961)
Supplement: S3 Fig — The distribution of ratios of the coalescence rate to the Yule rate sampled in the analysis. If the values are above 0, without negative values, then the model is a good approximation of the reality of the data. In reference to Fig 2. (DOCX) [file pone.0256961.s003.docx]

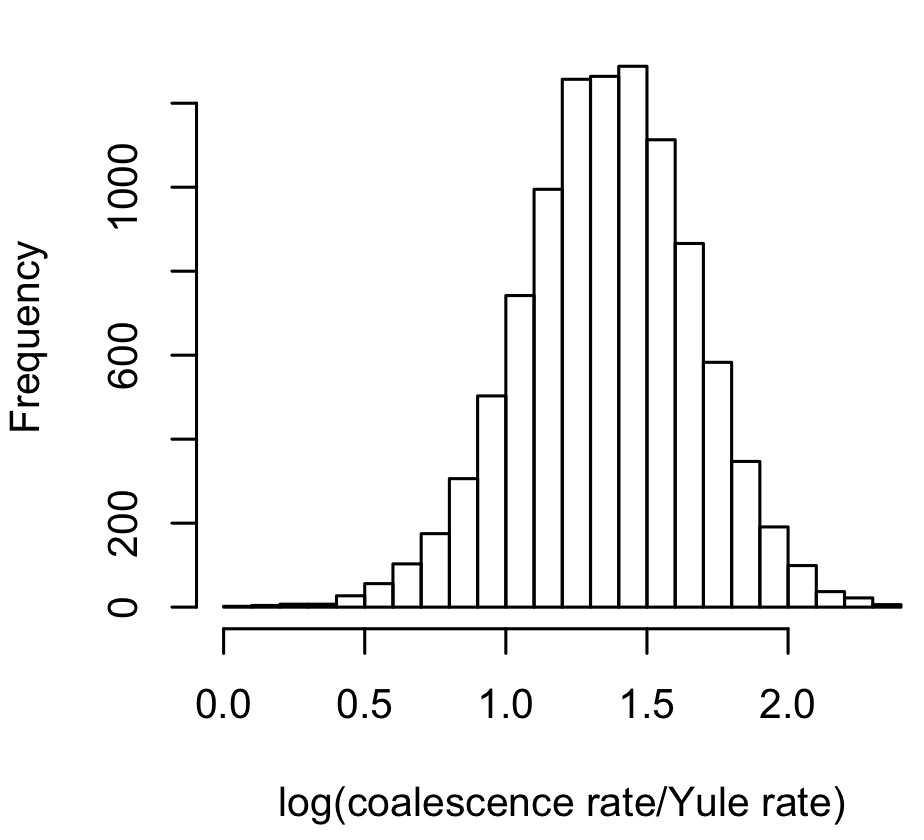


**S4 Fig.** **bGMYC analyses.** The distribution of ratios of the coalescence rate to the Yule rate sampled in the analysis. If the values are above 0, without negative values, then the model is a good approximation of the reality of the data. In reference to figure 2.
