# Supplementary material for: Blue mussels of the Mytilus edulis species complex from South America: The application of species delimitation models to DNA sequence variation
Source: PLoS One. 2021 Sep 2;16(9):e0256961. doi: 10.1371/journal.pone.0256961 (PMC8412288; doi:10.1371/journal.pone.0256961)
Supplement: S4 Fig — From the sequences of Fig 4. A) introduced Mytilus galloprovincialis in Chile, B) M. chilensis mussels from the Pacific and southern Atlantic coasts of South America, C) mussels from the Atlantic coast of South America. Scale bar: Genetic distance. (DOCX) [file pone.0256961.s004.docx]

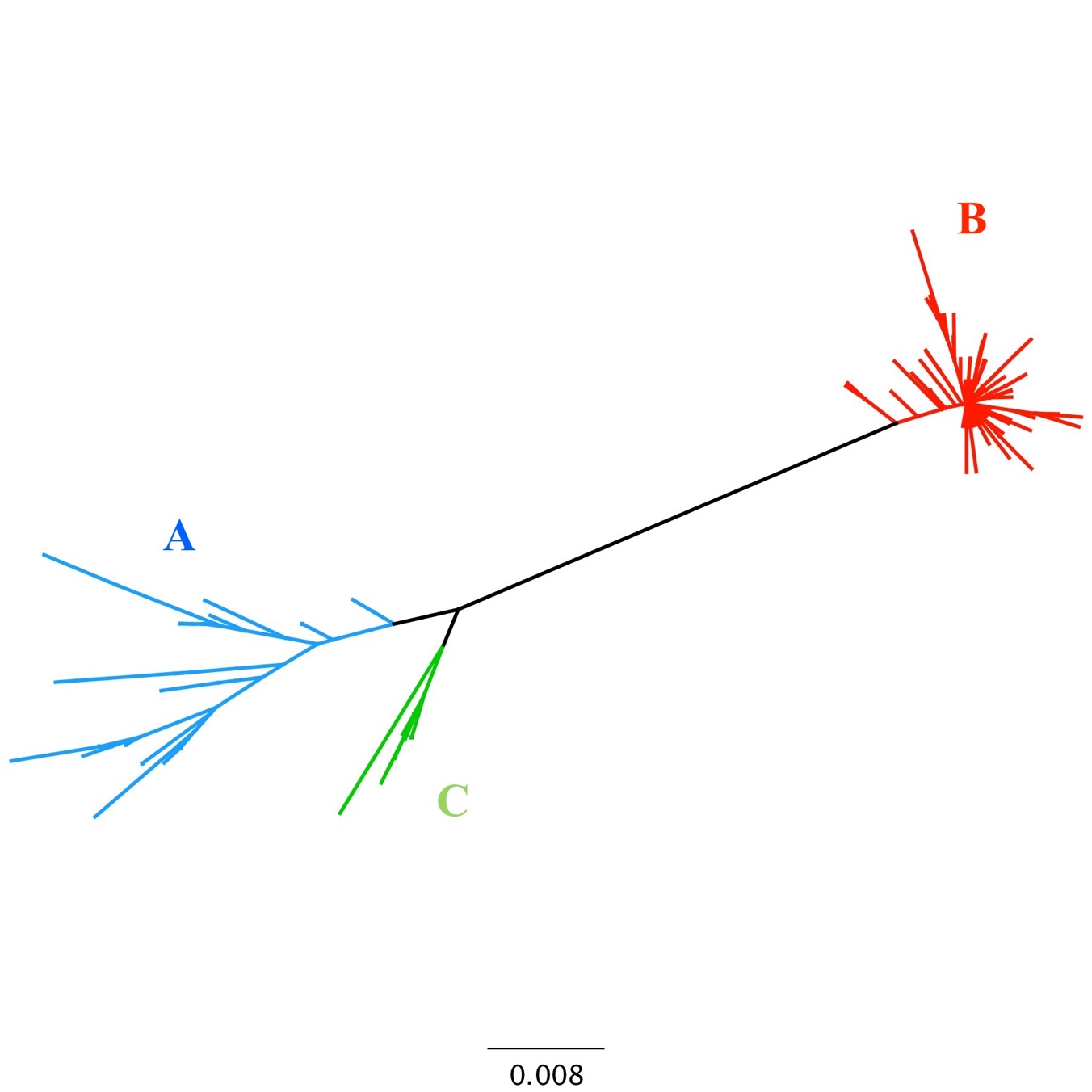


**S5 Fig.** **Unrooted tree drawn from neighbour-joining analysis.** From the sequences of figure 4. A) introduced *Mytilus galloprovincialis* in Chile, B) *M. chilensis* mussels from the Pacific and southern Atlantic coasts of South America, C) mussels from the Atlantic coast of South America. Scale bar = Genetic distance.
