# Supplementary material for: Blue mussels of the Mytilus edulis species complex from South America: The application of species delimitation models to DNA sequence variation
Source: PLoS One. 2021 Sep 2;16(9):e0256961. doi: 10.1371/journal.pone.0256961 (PMC8412288; doi:10.1371/journal.pone.0256961)
Supplement: S1 Table — Taxonomic index of congruence (Ctax) calculated for each pair of approaches. Mean of all the Ctax values obtained involving a given approach (Mean Ctax) and total number of species supported by each approach (sp.) is indicated. Species delimitation approaches: General Mixed Yule Coalescent model (GMYC), Bayesian General Mixed Yule Coalescent model (bGMYC; 0.95), Poisson Tree Processes (PTP), bayesian Poisson Tree Processes (bPTP), Bayesian Species Delimitation (BPP). (DOCX) [file pone.0256961.s005.docx]

**S2 Table**. **Taxonomic index of congruence (Ctax)**. Taxonomic index of congruence (Ctax) calculated for each pair of approaches. Mean of all the Ctax values obtained involving a given approach (Mean Ctax) and total number of species supported by each approach (sp.) is indicated. Species delimitation approaches: General Mixed Yule Coalescent model (GMYC), Bayesian General Mixed Yule Coalescent model (bGMYC; 0.95), Poisson Tree Processes (PTP), bayesian Poisson Tree Processes (bPTP), Bayesian Species Delimitation (BPP).

|  | **Paired Ctax** | | | | | **Mean Ctax** | **Species number** |
| --- | --- | --- | --- | --- | --- | --- | --- |
|  | **PTP** | **bPTP** | **GMYC single** | **bGMYC** | **BPP** |  |  |
| **PTP** | - |  |  |  |  | 0.89 | 8 |
| **bPTP** | 1.00 | - |  |  |  | 0.89 | 8 |
| **GMYC single** | 0.80 | 0.80 | - |  |  | 0.80 | 10 |
| **bGMYC** | 0.80 | 0.80 | 1.00 | - |  | 0.82 | 10 |
| **BPP** | 0.75 | 0.75 | 0.60 | 0.67 | - | 0.69 | 6 |
