# Supplementary material for: Blue mussels of the Mytilus edulis species complex from South America: The application of species delimitation models to DNA sequence variation
Source: PLoS One. 2021 Sep 2;16(9):e0256961. doi: 10.1371/journal.pone.0256961 (PMC8412288; doi:10.1371/journal.pone.0256961)
Supplement: S2 Table — GenBank accession numbers of sequence used in the analysis of Fig 1S for gen cytochrome c oxidase subunit I (COI). (DOCX) [file pone.0256961.s006.docx]

**S6 Table**. GenBank accession numbers of sequence used in the analysis of figure S1 for gen cytochrome *c* oxidase subunit I (COI).

| **Species** | **GenBank Accession** | **Species** | **GenBank Accession** |
| --- | --- | --- | --- |
| *Mytilus* sp. | AM905161 | *Mytilus* sp. | AM905224 |
| *Mytilus edulis* | AF241951 | *Mytilus edulis* | AY723898 |
| *Mytilus edulis* | AY723900 | *Mytilus galloprovincialis* | AY130060 |
| *Mytilus trossulus* | AY130064 | *Mytilus trossulus* | AY130066 |
| *Mytilus coruscus* | KC139309 | *Mytilus californianus* | MCU68776 |
| *Crenomytilus grayanus* | KM197988 | *Crenomytilus grayanus* | KM197989 |
| *Perna perna* | DQ917588 | *Perna perna* | DQ917594 |
| *Perna perna* | DQ917618 | *Perna canaliculus* | DQ917607 |
| *Perna canaliculus* | DQ917608 | *Perna canaliculus* | DQ917613 |
| *Perna viridis* | DQ917590 | *Perna viridis* | DQ917599 |
| *Perna viridis* | DQ917612 | *Mytella charrana* | KP013802 |
| *Mytella charruana* | KP013803 | *Mytella charruana* | KP013804 |
| *Choromytilus chorus* | JF301720 | *Choromytilus chorus* | JF301721 |
| *Choromytilus chorus* | JF301722 | *Choromytilus chorus* | JF301723 |
| *Musculus niger* | KF643542 | *Musculus niger* | KF644120 |
| *Musculus discors* | KR084526 | *Musculus discors* | KR084795 |
| *Crenella faba* | KF644051 | *Crenella faba* | KF644153 |
| *Crenella faba* | KF644170 | *Brachidontes pharaonis* | AY129566 |
| *Brachidontes pharaonis* | DQ836013 | *Brachidontes darwinianus* | KC844413 |
| *Brachidontes darwinianus* | KC844414 | *Brachidontes exustus* | AY621900 |
| *Brachidontes semilaevis* | AY825116 | *Brachidontes semilaevis* | AY825117 |
| *Brachidontes puniceus* | HM999788 | *Brachidontes puniceus* | HM999789 |
| *Semimytilus algosus* | JF301893 | *Semimytilus algosus* | JX891525 |
| *Semimytilus algosus* | KC107227 | *Brachidontes rodriguezii* | KC844483 |
| *Brachidontes rodriguezii* | KC844484 | *Perumytilus purpuratus* | KC844434 |
| *Perumytilus purpuratus* | KC844453 | *Ischadium recurvum* | AY621932-33 |
| *Geukensia demissa* | GDU56844 | *Geukensia demissa* | GQ282995 |
| *Brachidontes adamsianus* | AY825184 | *Mytilaster minimus* | DQ836022 |
| *Bathymodiolus septemdierum* | AB101429-30 | *Bathymodiolus azaricus* | AB170060-62 |
| *Bathymodiolus puteoserpentis* | HF545102 | *Bathymodiolus heckerae* | JN021264 |
| *Bathymodiolus brooksi* | JN021265 | *Bathymodiolus brooksi* | JN021266 |
| *Bathymodiolus heckerae* | JN021263 | *Gigantidas gladius* | AY608437 |
| *Gigantidas gladius* | AY649802 | *Bathymodiolus thermophilus* | GQ473901-02 |
| *Bathymodiolus antarcticus* | JN978652-53 | *Tamu fisheri* | AY649803 |
| *Tamu fisheri* | KM024269 | *Tamu fisheri* | KM024272 |
| *Adipicola longissima* | EU350072 | *Adipicola longissima* | HF545123 |
| *Idas* sp. | FJ158573 | *Idas* sp. | FJ158587 |
| *Adipicola pacifica* | AB257528 | *Adipicola pacifica* | AB539005 |
| *Bathymodiolus childressi* | AB257532 | *Bathymodiolus childressi* | AB257533 |
| *Benthomodiolus geikotsucola* | AB679346 | *Bathomodiolus lignicola* | AY275545 |
| *Modiolus areolatus* | DQ917604 | *Modiolus modiolus* | HM884246 |
| *Modiolus modiolus* | KR084900 | *Modiolus kurilensis* | KP243079 |
| *Modiolus auriculatus* | GQ480317 | *Xenostrobus pulex* | DQ917582 |
| *Xenostrobus atratus* | GQ480326 | *Xenostrobus atratus* | GQ480327 |
| *Xenostrobus securis* | JF430153 | *Xenostrobus securis* | JF430154 |
| *Modiolus barbatus* | KR084926 | *Modiolus barbatus* | KR084927 |
| *Lithophaga* sp. | KC706876 | *Aulacomya atra* | DQ917614 |
| *Aulacomya maorina* | DQ917615 | *Aulacomya atra* | JF301751/55/57 |
| *Crassostrea gigas* | KP099051-52 | *Modiolula phaseolina* | JF496770 |
| *Modiolus nipponicus* | AB076912 | *Mytilaster solidus* | JF496771 |
| *Septifer excisus* | AB076921-22 |  |  |
